# Cancer incidence in northern Sweden before and after the Chernobyl nuclear power plant accident

**DOI:** 10.1007/s00411-014-0545-6

**Published:** 2014-05-09

**Authors:** Hassan Alinaghizadeh, Martin Tondel, Robert Walinder

**Affiliations:** Occupational and Environmental Medicine, Department of Medical Sciences, Uppsala University, Uppsala, Sweden

**Keywords:** Cancer, Cesium-137, Chernobyl, Ecological study, Environment, Epidemiology, Ionizing radiation, Nuclear accident, Radiation, Sweden

## Abstract

Sweden received about 5 % of the total release of ^137^Cs from the Chernobyl nuclear power plant accident in 1986. The distribution of the fallout mainly affected northern Sweden, where some parts of the population could have received an estimated annual effective dose of 1–2 mSv per year. It is disputed whether an increased incidence of cancer can be detected in epidemiological studies after the Chernobyl nuclear power plant accident outside the former Union of Soviet Socialist Republics. In the present paper, a possible exposure–response pattern between deposition of ^137^Cs and cancer incidence after the Chernobyl nuclear power plant accident was investigated in the nine northernmost counties of Sweden (2.2 million inhabitants in 1986). The activity of ^137^Cs from the fallout maps at 1986 was used as a proxy for the received dose of ionizing radiation. Diagnoses of cancer (ICD-7 code 140-209) from 1980 to 2009 were received from the Swedish Cancer Registry (273,222 cases). Age-adjusted incidence rate ratios, stratified by gender, were calculated with Poisson regression in two closed cohorts of the population in the nine counties 1980 and 1986, respectively. The follow-up periods were 1980–1985 and 1986–2009, respectively. The average surface-weighted deposition of ^137^Cs at three geographical levels; county (*n* = 9), municipality (*n* = 95) and parish level (*n* = 612) was applied for the two cohorts to study the pre- and the post-Chernobyl periods separately. To analyze time trends, the age-standardized total cancer incidence was calculated for the general Swedish population and the population in the nine counties. Joinpoint regression was used to compare the average annual percent change in the general population and the study population within each gender. No obvious exposure–response pattern was seen in the age-adjusted total cancer incidence rate ratios. A spurious association between fallout and cancer incidence was present, where areas with the lowest incidence of cancer before the accident coincidentally had the lowest fallout of ^137^Cs. Increasing the geographical resolution of exposure from nine county averages to 612 parish averages resulted in a two to three times higher value of variance in the regression model. There was a secular trend with an increase in age-standardized incidence of cancer in both genders from 1980 to 2009, but significant only in females. This trend was stronger and statistically significant for both genders in the general Swedish population compared to the nine counties. In conclusion, using both high quality cancer registry data and high resolution exposure maps of ^137^Cs deposition, it was not possible to distinguish an effect of ^137^Cs on cancer incidence after the Chernobyl nuclear power plant accident in Sweden.

## Introduction

It is disputed whether cancer from ionizing radiation can be detected in epidemiological studies on cohorts exposed to radioactive releases from the Chernobyl nuclear power plant accident, outside the former Union of Soviet Socialist Republics (USSR). The main argument is that the low doses associated with those releases represent a tiny contribution to the total risk of developing cancer, and thereby is obscured by several other more prominent non-radiation risk factors for cancer.

In April 26th 1986 an accident occurred at the Chernobyl nuclear power plant in Ukraine, USSR, and a large amount of radioactive material was released with the highest ground deposition of nuclides in Belarus, Russia and Ukraine. In Belarus an increase in thyroid cancer incidence in children was seen in 1990, that later was confirmed to be related to the Chernobyl nuclear power plant accident (UNSCEAR 2000). An overall evaluation by an expert group assigned by the WHO has concluded that apart from the large increase in the thyroid cancer incidence, there is no clearly demonstrated radiation-related increased cancer risk in the former USSR (Cardis et al. 2006).

Sweden received a relatively large amount of the total radioactive fallout (about 5 % of the total released ^137^Cs) from the Chernobyl nuclear power plant accident (Mattsson and Moberg 1991). The main contributors to the dose rate in the first weeks were short-lived nuclides, later replaced by the long-lived ^134^Cs and ^137^Cs. The average dose rate to the Swedish population has been estimated to less than 0.1 mSv per year, but certain risk groups such as reindeer herders could have received an annual dose of 1–2 mSv per year following the accident (Moberg and Reizenstein 1993). According to the same report an estimated 300 extra cases of cancer deaths could be attributed to the fallout in Sweden during 50 years after the Chernobyl nuclear power plant accident if the linear-no-threshold (LNT) hypothesis is applied. This number compares to about 1 million spontaneous cancer deaths that are expected to occur among the Swedish population in 50 years (based on current spontaneous cancer rates), due to reasons other than exposure to the Chernobyl fallout. The average received dose from the Chernobyl fallout in Sweden is less than the dose from the terrestrial gamma radiation (estimated to be 1–5 mSv per year) and cosmic radiation (<0.5 mSv per year) (Andersson et al. 2007). Nevertheless, there has been a public concern, especially in the regions in Sweden with the highest fallout, and also awareness among authorities, that the accident might have some health impacts on the population. Therefore, a food regulation program was introduced in 1986 with a maximum allowed activity in food sold to the public of 300 Bq ^137^Cs per kilogram to keep the dose from food intake below 1 mSv per year. In 1987 a new limit of 1,500 Bq per kilogram was introduced for game and reindeer meat, wild berries, mushrooms, fresh water fish and nuts sold to the public (Persson and Prethun 2002).

A long latency period between the exposure of ionizing radiation and the development of cancer makes the contribution of other risk factors more prominent, such as life-style, food habits or chemical exposure. Age is the most important personal risk factor associated with cancer and therefore regional differences in age distribution can sometimes explain spatial differences in cancer incidence. In Sweden there is a well-known secular trend, with crude rates of total cancer incidence in Sweden increasing about 2 % each year in the last decades (SCB 2012; EpC 2012). Hypothetically, a trend shift could be expected in the population if the radiation dose influenced the cancer incidence after a latency period of less than 5 years for leukemia and 10–20 years for solid tumors (BEIR VII 2006). However, it might be misleading to use specific time-windows for latency periods of radiation-induced cancer when exposure from contaminated ground is present for decades, in contrast to a situation with an acute single exposure. Note that after 5 years, and only taking the physical decay into consideration, the physical activity of ^137^Cs is still 85 % of the initial activity in some contaminated soil and, to some extent present in the foodstuffs. Moreover, the knowledge of latency periods is mainly based on one short-term exposure of the Life Span Study (LSS) cohort of atomic bomb survivors in Japan (Preston et al. 2007; Richardson et al. 2009). According to a previous epidemiological study, an early increase in the incidence of total cancer related to the fallout of ^137^Cs was noticed in Sweden already a few years after the Chernobyl nuclear power plant accident, suggesting an early promoting effect (Tondel et al. 2006). Therefore, with the large range of different latency periods for different cancers and the prolonged exposure, together with earlier epidemiological findings, we chose to omit the presentation of different time-windows. Instead, only one follow-up period was used in the present study protocol.

In a previous epidemiological study 1,278 incident cases of cancer could be calculated as attributed to the fallout in Sweden during a follow-up period from 1988 to 1999, unexpectedly high taking the low dose and the short latency period into account (Tondel et al. 2006). A similar study has been performed in Finland showing no association between cancer incidence and fallout of ^137^Cs when comparing the cancer incidence before (1981–1985) and after (1988–2007) the Chernobyl nuclear power plant accident (Kurttio et al. 2013).

The present study was restricted to those counties with the highest fallout of ^137^Cs in Sweden, but it also included less exposed counties serving as reference areas. This restriction can also be justified to obtain a somewhat more homogeneous population regarding trades, life-style, hospital admission and the environment, by excluding larger urban and agricultural areas in the south of Sweden. In the present paper the term cancer is used, equivalently to malignancies (including leukemia).

As a proxy for the absorbed dose the ground deposition of ^137^Cs is used in the present paper, and the assumption that individuals living in more contaminated areas receive higher doses, both regarding external radiation dose from the ground and internal dose from locally produced contaminated food.

The main hypothesis of the present study was that an exposure–response in cancer incidence could be identified after the Chernobyl nuclear power plant accident, but it should also be explored whether such response could be influenced by ecological bias through aggregation of data on different geographical levels: parish, municipality or county. Thus, an ecological study design was chosen, with ^137^Cs exposure at group level in association with cancer at the individual level. Furthermore, the pre-Chernobyl regional differences in cancer incidence should be studied, to investigate if such regional differences were present before the accident in 1986, hence acting as a potential confounding factor.

## Materials and methods

### Study design

The study design is partial ecological with cross-level analysis of cancer incidence on the individual level and environmental exposure assessment (calculated average deposition of ^137^Cs) on a group level: county, municipality and parish, respectively. A comparison of the cancer incidence before and after the Chernobyl nuclear power plant accident (April 26th 1986, with fallout reaching Sweden on April 28th) was made by creating two closed cohorts, before and after the accident. Both cohorts consisted of the population in the nine northernmost out of totally 21 counties in Sweden: Norrbotten, Västerbotten, Jämtland, Västernorrland, Gävleborg, Dalarna, Västmanland, Uppsala and Södermanland (2.2 million people in 1986). Personal identification numbers of the population in the two cohorts were retrieved from the National Archives of Sweden. The two closed cohorts were defined as subjects alive any time from January 1st to December 31th in 1980 for the pre-Chernobyl cohort and from April 28th to December 31st 1986 for the post-Chernobyl cohort. The start of the follow-up period of cancer incidence was January 1st 1980 for the pre-Chernobyl cohort and April 28th 1986 for the post-Chernobyl cohort. The follow-up period ended December 31st 1985 (up to 6 years) for the pre-Chernobyl cohort and December 31st 2009 (up to 23.7 years) for the post-Chernobyl cohort.

Number of person-years was calculated for each subject of the two cohorts until the first diagnosis of cancer and censored by the date of death or the end of the follow-up periods, respectively.

### Cancer cases

All cases of cancer (ICD-7 code 140-209) with the date of diagnosis and the date of deaths (all causes) were retrieved from the start of the registry at the National Board of Health and Welfare from January 1st 1958 to December 31st 2009. In total 368,244 cases of cancer were identified. Subjects with a diagnosis of cancer prior to start of the follow-up periods were excluded from the cohorts, and in cases of multiple cancer diagnoses only the first diagnosis of cancer was considered during the follow-up period, since multiple cancers in each subject might not be independent events.

### Exposure to ^137^Cs

Data of the surface-weighted average deposition of ^137^Cs for each county (*n* = 9), municipality (*n* = 95) and parish (*n* = 612) were received from the Swedish Radiation Safety Authority and ranged from 2 to 28; 2 to 58; and 1 to 85 kBq/m^2^, respectively, excluding water areas when calculating these averages. The areas with the lowest fallout in 1986 were used as the reference category (≤2.6 kBq/m^2^) for both the period 1980–1985 and 1986–2009. By assignment from the Swedish Radiation Safety Authority, the Geological Survey of Sweden had performed yearly aerial gamma-radiation measurements of Sweden (gamma-spectrum of ^137^Cs, by a special equipped Cessna 240 flying at a height of 30 or 60 m above the ground with a line spacing of 200–5,000 m). These measurements were stored in a database with results given in kBq/m^2^ of ^137^Cs in a 200 × 200 meter grid map backdated to May 1st 1986 (in total 9.9 million measurement points) and provided to the Swedish Radiation Safety Authority for calculation of county, municipality and parish averages, respectively. In order to test if an exposure–response pattern existed prior to the Chernobyl nuclear power plant accident in 1986, the same surface-weighted value for each parish, municipality and county was used for both the 1980 cohort and 1986 cohort. This means that a fictive fallout map was applied for the 1980 cohort, using the map from May 1st 1986 when classifying the exposure for the subjects in both cohorts.

### Statistical methods

The annual population for each county was retrieved from Statistics Sweden (SCB 2012) while the annual number of total cancer cases was retrieved from the National Board of Health and Welfare (Cancer incidence in Sweden - 2011 2012). Time trend analyses of age standardized cancer incidence during the study period (1980–2009) were made by the annual average percent change (AAPC) method using Joinpoint Regression Program, Version 4.0.4. May 2013; Statistical Research and Applications Branch, National Cancer Institute (Kim et al. 2000). The Swedish standard population from year 2000 in 5 years age categories, was used for the age standardized total cancer incidence.

Poisson regression was used to follow the incidence rates over time. Due to the overestimation of error for the estimated relative risk or incidence rate ratio (IRR), with the lowest exposure category as a reference category, (≤2.6 kBq/m^2^), the Poisson regression was adjusted by using a procedure known as sandwich estimation (Royall 1986). Five exposure categories were created based on the average county deposition of ^137^Cs in order to analyze a possible exposure response pattern. The same exposure categories were also applied for municipalities and parishes. Stratified on gender the age-adjusted cancer incidence rate ratios were calculated for the three geographical levels separately. A possible ecological bias from geographical exposure misclassification was visualized by comparing sigma-u values for the three geographical levels: county (*n* = 9), municipality (*n* = 95) and parish level (*n* = 612). Sigma-u is the variance of the residuals of the IRR at each geographic level. A higher variance is interpreted as a higher degree of explanation of the IRR at that geographical level.

All calculations were performed by using SAS version 9.3 (SAS Institute Inc., Cary, NC) and Stata version 13.0 (StataCorp LP, College Station, Texas, USA).

## Results

Descriptive data of the number of individuals, number of cancer cases and person-years and of cancer cases for the pre- (1980) and the post-Chernobyl (1986) cohorts are presented in Table 1. For the 1986 cohort the mean age with standard deviation at inclusion was 38 ± 23 years for males and 40 ± 24 years of age for females. An overall age at the end of follow-up period (31st December, 2009) for males was 58 ± 19 years and for females 60 ± 20 years of age.

**Table 1:** Descriptive data of the two cohorts in the nine northernmost counties of Sweden. Exposure to ^137^Cs on the ground in 1986 (kBq/m^2^) applied for the two cohorts and divided in five categories at three geographical levels: county, municipality and parish level

Cesium-137 (kBq/m^2^)	% of residence^a^ (number of areas)	Number of individuals 1980 (number of cancer cases 1980–1985) Person years during 1980–1985		Number of individuals 1986 (number of cancer cases 1986–2009) Person years during 1986–2009	
		Males 1,106,320 (26,114)	Females 1,102,054 (24,641)	Males 1,111,607 (115,770)	Females 1,115,104 (106,697)
*9 counties*					
≤2.6	24.4 (2)	274,153 (5,854)	269,639 (5,724)	272,700 (26,357)	270,470 (24,168)
		1,675,532	1,654,734	5,490,814	5,485,400
2.7–6.5	17.2 (2)	190,647 (4,987)	189,914 (4,371)	191,154 (20,802)	191,134 (19,157)
		1,162,393	1,163,007	3,815,807	3,844,825
6.6–14.0	24.3 (2)	272,139 (6,488)	271,263 (6,186)	270,107 (28,659)	271,051 (26,469)
		1,661,100	1,662,543	5,409,600	5,455,767
14.1–28.3	22.4 (2)	237,924 (5,418)	239,322 (5,271)	247,809 (25,796)	251,604 (23,974)
		1,456,493	1,470,597	5,058,239	5,179,549
28.4	11.7 (1)	131,457 (3,367)	131,916 (3,089)	129,837 (14,156)	130,845 (12,929)
		800,296	806,726	2,577,981	2,606,949
*95 municipalities*					
≤2.6	24.2 (34)	274,850 (5,934)	267,555 (5,585)	271,513 (26,462)	266,869 (23,992)
		1,679,881	1,642,770	5,466,541	5,416,687
2.7–6.5	21.8 (21)	240,743 (5,794)	241,383 (5,545)	241,588 (25,292)	243,447 (23,681)
		1,468,105	1,478,181	4,830,458	4,900,772
6.6–14.0	23.0 (14)	245,962 (5,944)	248,370 (5,708)	253,658 (26,758)	257,433 (25,093)
		1,505,036	1,524,164	5,152,117	5,256,137
14.1–28.3	18.8 (17)	208,525 (4,824)	207,259 (4,513)	209,525 (22,384)	210,071 (20,160)
		1,274,134	1,271,944	4,220,914	4,269,672
28.4–51.8	12.2 (9)	136,240 (3,618)	137,487 (3,290)	135,323 (14,874)	137,284 (13,771)
		828,658	840,548	2,682,412	2,729,222
*612 parishes*					
≤2.6	32.5 (152)	362,066 (8,030)	357,827 (7,776)	362,418 (36,218)	360,688 (33,367)
		2,213,311	2,196,023	7,299,538	7,309,831
2.7–6.5	18.6 (137)	208,621 (5,070)	206,310 (4,597)	207,273 (21,812)	206,571 (19,979)
		1,272,162	1,264,392	4,147,623	4,169,434
6.6–14.0	16.6 (118)	178,450 (4,331)	179,471 (4,153)	184,165 (19,273)	186,033 (18,052)
		1,090,347	1,099,562	3,721,379	3,779,249
14.1–28.3	18.0 (126)	196,732 (4,464)	195,476 (4,249)	200,666 (21,254)	201,352 (19,160)
		1,203,077	1,200,974	4,065,555	4,117,376
28.4–85.3	14.3 (79)	160,451 (4,219)	162,970 (3,866)	157,085 (17,213)	160,460 (16,139)
		976,917	996,655	3,118,347	3,196,599

^a^Percent of study population 1986 in each cesium-category

**Fig. 1:**
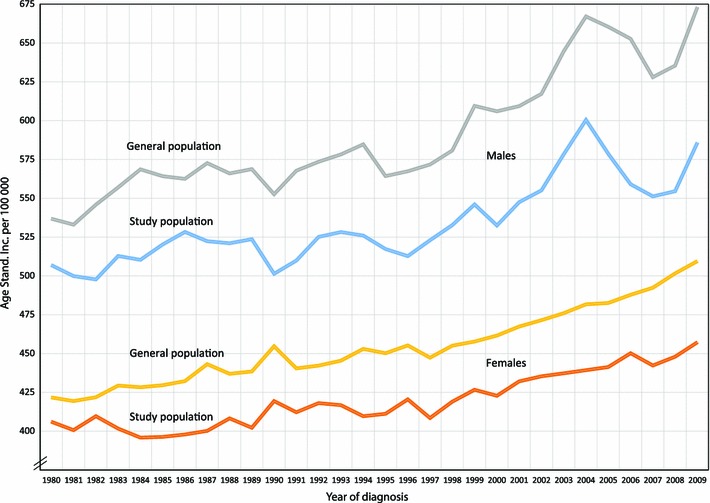
Age-standardized incidence of total cancer per 100,000 from 1980 to 2009. Rates are age-adjusted to the 2000 Swedish standard population by 5-year age groups

The age-standardized total cancer incidence per 100,000 (males and females, separately) from 1980 to 2009 in the study population and the general population is presented in Fig. 1 with trends in Table 2. The joinpoint regression resulted in a three point model for males and a one point model for females. For males a significant annual percent change was found for the period 1997–2004 in the general population and for the periods 1980–2000, but also 2000–2004 in the study population. For females significant changes were found for the periods 1980–2000 and 2000–2009 in the general population. In the female study population there were non-significant changes during 1980–1985 but a significant change 1985–2009. For the whole study period (1980–2009) females had a significant AAPC in both general- and study populations (AAPC = 0.6; 0.5–0.7 resp. AAPC = 0.3; 0.2–0.5), but for males only a significant change in the general population (AAPC = 0.7; 0.3–1.1) was found, and not in the study population (AAPC = 0.5; −0.2 to 1.2).

**Table 2:** Trends in percent of age-standardized cancer incidence during 1980–2009 by gender

	Trend 1		Trend 2		Trend 3		Trend 4		Full range (1980–2009)
	Years	APC (95 % CI)	Years	APC (95 % CI)	Years	APC (95 % CI)	Years	APC (95 % CI)	APPC (95 % CI)
*General population*									
Males	(1980–1984)	1.5 (−0.2–3.3)	(1984–1997)	0.1 (−0.2–0.4)	(1997–2004)	1.9 (1.0–2.8)*	(2004–2009)	−0.1 (−1.4–1.1)	0.7 (0.3–1.1)*
Females	(1980–2000)	0.4 (0.4–0.5)*	(2000–2009)	1.0 (0.8–1.3)*					0.6 (0.5–0.7)*
*Study population*									
Males	(1980–2000)	0.2 (0.1–0.4)*	(2000–2004)	2.9 (0.2–5.6)*	(2004–2007)	−2.9 (−7.8–2.3)	(2007–2009)	3.2 (−2.0–8.8)	0.5 (−0.2–1.2)
Females	(1980–1985)	−0.5 (−1.4–0.4)	(1985–2009)	0.5 (0.4–0.6)*					0.3 (0.2–0.5)*

Trend years may include different time periods based on joinpoint regression modeling

*AAPC* Average annual percent change, full range (1980–2009), *APC* annual percent change, *CI* confidence interval

* Significant different from zero at alpha = 0.05

Analysis in terms of the five exposure categories could not reveal any obvious exposure–response pattern in age-standardized cancer incidence rate ratios, neither in the pre- (1980–1985), nor in the post-Chernobyl (1986–2009) follow-up periods (Table 3). The reference region (≤2.6 kBq/m^2^) had the lowest cancer incidence rate both before and after 1986 for both genders. In the highest exposure category (≥28.4 kBq/m^2^) the cancer IRR decreased for males after the Chernobyl nuclear power plant accident compared to before at county, municipality and parish level. In contrary, for females there were increased IRR at all exposure categories comparing the two cohorts 1980 and 1986, respectively (except in one category; 6.6–14.0 kBq/m^2^ at municipality level). Sub-analyses of IRRs among two specific radiation sensitive malignancies, thyroid cancer and leukemia, and among children aged 0–20 years, did not reveal any exposure–response patterns (data not shown).

**Table 3:** Age-adjusted cancer incidence rate ratios (IRR) stratified by gender with 95 % CI in brackets during the pre- (1980–85) and post- (1986–2009) Chernobyl periods for five exposure categories to ^137^Cs (kBq/m^2^) at three geographical levels

Cesium-137 (kBq/m^2^)	Counties (*n* = 9)		Municipalities (*n* = 95)		Parishes (*n* = 612)	
	1980–1985	1986–2009	1980–1985	1986–2009	1980–1985	1986–2009
*Males*						
≤2.6	(Ref)	(Ref)	(Ref)	(Ref)	(Ref)	(Ref)
2.7–6.5	1.153 (1.111–1.198)	1.114 (1.094–1.134)	1.047 (1.010–1.086)	1.059 (1.041–1.077)	1.041 (1.001–1.079)	1.049 (1.032–1.067)
6.6–14.0	1.087 (1.050–1.126)	1.090 (1.072–1.109)	1.144 (1.104–1.186)	1.127 (1.108–1.147)	1.079 (1.040–1.120)	1.078 (1.059–1.097)
14.1–28.3	1.127 (1.086–1.170)	1.151 (1.131–1.170)	1.061 (1.021–1.102)	1.112 (1.093–1.132)	1.037 (1.000–1.076)	1.100 (1.082–1.119)
28.4+	1.101 (1.055–1.149)	1.093 (1.071–1.116)	1.108 (1.062–1.155)	1.100 (1.079–1.123)	1.094 (1.054–1.135)	1.071 (1.051–1.090)
Sigma-u	0.06 (0.04–0.10)	0.04 (0.02–0.06)	0.09 (0.08–0.12)	0.07 (0.06–0.09)	0.10 (0.09–0.12)	0.08 (0.07–0.09)
*Females*						
≤2.6	(Ref)	(Ref)	(Ref)	(Ref)	(Ref)	(Ref)
2.7–6.5	1.043 (1.003–1.085)	1.114 (1.093–1.135)	1.054 (1.015–1.094)	1.074 (1.055–1.093)	1.007 (0.971–1.045)	1.047 (1.028–1.065)
6.6–14.0	1.044 (1.007–1.082)	1.078 (1.060–1.097)	1.111 (1.071–1.153)	1.108 (1.089–1.128)	1.058 (1.020–1.099)	1.073 (1.053–1.092)
14.1–28.3	1.098 (1.058–1.140)	1.117 (1.098–1.138)	1.050 (1.009–1.091)	1.082 (1.062–1.103)	1.040 (1.002–1.080)	1.062 (1.043–1.081)
28.4+	1.026 (0.982–1.072)	1.078 (1.055–1.101)	1.044 (1.000–1.090)	1.092 (1.070–1.116)	1.016 (0.978–1.056)	1.066 (1.046–1.086)
Sigma-u	0.04 (0.02–0.07)	0.02 (0.01–0.03)	0.06 (0.04–0.08)	0.04 (0.03–0.05)	0.06 (0.05–0.09)	0.05 (0.04–0.06)

*Sigma-u* standard deviation of residual within groups (county, municipality and parish, respectively) indicating the degree of variance

An increased resolution in exposure assessment from nine areas (county level); to 95 areas (municipality level), or to 612 areas (parish level) did not reveal any apparent differences in exposure–response pattern (Table 3). Sigma-u increased for both males and females (from 4 to 8 % and from 2 to 5 %, respectively) in the post-Chernobyl follow-up period (1986–2009) when the geographical exposure resolution increased from nine counties to 612 parishes (Table 3).

## Discussion

There is a tendency for a secular trend with an increase in age-standardized incidence of total cancer from 1980 to 2009. The trend is significant during the whole period in the general population of Sweden for both males and females, whereas only significant among females in the study population (Fig. 1). Nationwide screening activities; cervix smear tests introduced in 1960ies and mammography in 1980ies cannot explain the continuous increase and would also only influence the female incidence. Other possible factors explaining the increasing incidence include development of new diagnostic procedures for cancer, changes in autopsy frequency, demographic changes, urbanization, increased medical use of X-ray, life-style changes (i.e. UV-radiation exposure, more sedentary work, changed food habits and increased body weight) or in various other environmental exposures such as infectious agents, air and water pollution, increased production of and introduction of new chemicals.

Cancer incidence was—during the study period—lower in the chosen nine counties compared to that of the general Swedish population. It is well-known that rural areas in Sweden have lower total incidence rates than urban areas (EpC 2012). The restriction in the present study to investigate the population of the northern part of Sweden—in an effort to avoid the influence from less well defined risk factors associated with an urban life-style and lower cesium exposure in south of Sweden—was therefore justified in order to avoid some potential confounding.

An exposure–response pattern of increased cancer incidence with higher deposition of ^137^Cs could not be revealed in the present study. Since the reference category had the lowest incidence rates, both before and after the accident in 1986, the areas with the lowest incidence rates before the accident coincidentally had the lowest fallout, irrespectively of geographical level. This spurious association between the lowest deposition and low pre-Chernobyl incidence rates could cause confounding if an adjustment for baseline incidence is not performed. Differences in cancer screening programs and registration of cancer have been suggested in a Finnish study as partly explaining regional differences in cancer incidence after the Chernobyl nuclear power plant accident. Breast cancer, which is associated with radiation (BEIR VII 2006) and the most common malignancy among women (EpC 2012), was therefore omitted from their analysis because of a supposed registration bias due to assumed local differences in diagnostic procedures of mammography (Kurttio et al. 2013). The Swedish health care system is divided in 21 county councils with independent budgets and health care organizations with subsequent regional differences in health care systems together with differences in the number of doctors per capita. Therefore, geographical differences, existing pre-Chernobyl, might be necessary to adjust for in future studies. Another possibility could be to stratify the analyses by county, if the exposure contrast (regional differences in the activity of ^137^Cs) and statistical power could be remained.

The present study, without a clear exposure–response trend, contrasts a previous study on the total cancer incidence in northern Sweden, where six exposure categories were used, having a greater exposure contrast, which might have rendered a higher sensitivity to identify a trend (Tondel et al. 2006). Exposure categories in the present study were created in an attempt to classify the exposure into quintiles for analyses of exposure-response trends. Exposure categorization was based on the average deposition of ^137^Cs in the counties, thereby setting the limitations for classifying the exposure on municipality and parish levels. We could use two counties in each exposure category, except the for the highest exposure category where only one county remained. As Västernorrland had the highest average county deposition of ^137^Cs (28.4 kBq/m^2^), this county constrained the upper category to 14 % of the study population for the parishes, and therefore reduced the exposure contrast for the parishes. Hence, there is a possibility of obfuscating an intra-categorical variation in exposure–response relationship for the parish classification. Yet, another consequence of having exposure categories based on the county averages is that the reference category for the parish level analyses became relatively large (one third of the population on the parish level) reducing the contrast of exposure even more. However, for the municipality level the categories of exposure almost remained in quintiles. In order to study a possible ecological bias fixed categories were used in the present study to allow comparison between all three geographical levels. There could have been an exposure misclassification due to the ecological design of the study using average exposure of ^137^Cs in rather large geographic areas, from county, to municipality down to parish level. In the present study it seems that an increased resolution of the exposure map from county to parish level, measured by the sigma-u value increasing twofold to threefold in the regression models, could reduce exposure misclassification i.e. reducing the ecological bias. An improved exposure classification could be achieved by having dwelling coordinates for all individuals matched to the ^137^Cs activity map. Another concern in exposure misclassification is that the exposure after 1986 is not considered, and thereby ignoring changes in cumulative exposure if people moved between areas during the follow-up period.

Another limitation of the present study is that only adjustment for age as a confounding factor was done in the analyses, because it was decided to stratify rather than adjust for gender. In contrast, the previous study (Tondel et al. 2006) only included people of ages 0–60 years at the time of the accident and might therefore have been more sensitive to see a relative increase in cancer incidence. Hence, stratifying for age groups would have been desirable in the present study. However, a sub-analysis of children aged 0–20 years in the present study did not reveal any tendency of exposure–response; neither did sub-analyses of the specific malignancies thyroid cancer and leukemia.

Moreover, data in the present study was only adjusted for age, whereas Tondel et al. (2006) made adjustments for population density, lung cancer incidence, pre-Chernobyl cancer incidence and terrestrial gamma radiation. Therefore, an obvious exposure–response trend can theoretically be obscured by negative confounding of these or other unidentified confounding factors. On the other hand, the previously observed exposure–response trend could have been explained by remaining and unadjusted geographical confounding, only including 2 years pre-Chernobyl cancer rates. Note that the present study did not control for radon exposure as a potential confounding factor since there are no comprehensive data on the spatial distribution of radon exposure in Sweden. However, it is believed that this is of less importance for the incidence of total cancer because lung cancer only contributed 6 % of the total number of cancer cases, in the present study.

For the present register-based study one can only speculate how food and smoking habits, various socioeconomic factors and any population density factor could correlate with the fallout of ^137^Cs. However, in a previous study, cancer was stratified into three categories (clearly, suspected and unrelated to smoking), which all had similar exposure–response patterns, indicating only a weak confounding effect from tobacco smoking (Tondel et al. 2004).

In the present study a ^137^Cs activity map from the year 1986 was used for the exposure assessment as a proxy for exposure to ionization radiation following the Chernobyl nuclear power plant accident. The small remaining fallout from the global nuclear weapons tests from the 1950ies and 1960ies was neglected as being low and uniformly distributed. A serious limitation could be that the fallout of ^137^Cs did not reflect the total individual exposure because the contribution to the dose from the short-lived nuclides (such as, for example, ^131^I, ^133^I, and ^134^Cs) that contributed to the exposure during the first years was ignored. Algorithms have been developed for the calculation of absorbed dose from the activity of ^137^Cs on the ground. The maximum recorded ^137^Cs activity in the study region (in a 200 meter grid cell), 165 kBq/m^2^ in the municipality of Gävle, would—according to these algorithms—correspond to a received external absorbed dose of 3.6 mGy from ^137^Cs during the first year, though assuming minimal shielding from building materials and seasonal snow cover (Finck 1992).

But only considering the external dose from the ground would underestimate the total absorbed dose since a significant contribution is also received from internal contamination from food. One way of calculating the internal dose could be using transfer factors based on whole body measurements of various population groups (Rääf et al. 2006). This could be especially important for families of reindeer herders, farmers, hunters and people with habits of high consumption of wild mushroom, berries and fresh water fish retrieved near their homes. The fact that—at the time of the Chernobyl nuclear power plant accident—Sweden still had dairies in each county probably has influenced the exposure and subsequent the cancer incidence on a county level. Therefore it might have obfuscated some parish effect on the cancer incidence because milk products were produced with milk from all over the county, rather than locally produced in the parishes.

Though, the dose of ionization radiation from the Chernobyl fallout is on average relatively low compared to other sources of ionizing radiation such as medical use, terrestrial- and cosmic radiation in Sweden (UNSCEAR 2011; IARC 2012; Andersson et al. 2007), we consider the data quality in the present study sufficient to draw some conclusions about cancer incidence. The follow-up period in the present study, 24 years, might still be too short to demonstrate a potential increase of many solid cancers, because of potentially long latency periods and also because of an ongoing exposure of people living in contaminated areas. Due to the long physical half-life of ^137^Cs of 30.2 years, 50 % of the activity is still remaining after 30 years. On the other hand, the study population is relatively large, 2.2 million individuals, which is the total population in about two thirds of the Swedish land area resulting in a fairly good statistical power to identify a small increased risk, if existent. The design of the present study is partly ecological with diagnoses of cancer at an individual level, but exposure at group level, from county to municipality and parish regarded as the smallest area. However, caution should be addressed when making conclusions about individual health risks when using a partial ecologic design with cross-level analysis, as in our case, since the design is particularly vulnerable to bias (Morgenstern 1998). One strength of the present study is that the cancer diagnoses retrieved from the national cancer registry data are, by international comparison, of high quality (Barlow et al. 2009). The high resolution fallout maps (200 × 200 m grid, with almost 10 million measurement points) give a fairly high accuracy of ^137^Cs deposition on a parish level, and include a relatively broad range in exposure from 2 to 85 kBq/m^2^. In order to increase the precision in future studies the exposure misclassification could be reduced by assessing both external and internal dose. The external dose could use more detailed mapping of both ^137^Cs from the Chernobyl accident and terrestrial gamma radiation by using individual dwelling coordinates over time to have more precise absorbed dose assessments.

## Conclusion

In conclusion, the present study, although using both high quality cancer registry data and high resolution exposure maps of ^137^Cs deposition, cannot distinguish the effect of ionizing radiation on cancer incidence in Sweden after the Chernobyl nuclear power plant accident—if there is any—from the natural variation in cancer incidence or influence from other possible risk factors.
